# Gender and Drug-Resistant Tuberculosis in Nigeria

**DOI:** 10.3390/tropicalmed8020104

**Published:** 2023-02-06

**Authors:** Olanrewaju Oladimeji, Bamidele Paul Atiba, Felix Emeka Anyiam, Babatunde A. Odugbemi, Tolulope Afolaranmi, Ayuba Ibrahim Zoakah, C. Robert Horsburgh

**Affiliations:** 1 Department of Public Health, Faculty of Health Sciences, Walter Sisulu University, Mthatha 5117, South Africa; 2 Department of Community Health, Faculty of Clinical Sciences, College of Health Sciences, University of Jos, Jos 2064, Nigeria; 3 Faculty of Health Sciences, Durban University of Technology, Durban 4001, South Africa; 4 Departments of Community Health & Primary Health Care, Lagos State University College of Medicine, Ikeja 102212, Nigeria; 5 Section of Infectious Diseases, Boston Medical Center, Boston, MA 02118, USA; 6 Department of Biostatistics, School of Public Health, Boston University, Boston, MA 02118, USA; 7 Department of Global Health, School of Public Health, Boston University, Boston, MA 02118, USA

**Keywords:** drug-resistant TB, gender, treatment zone, human immunodeficiency virus

## Abstract

We conducted a retrospective study of 2555 DR-TB patients admitted to treatment between 2010 and 2016 in six geopolitical zones in Nigeria. We characterized the gender distribution of DR-TB cases and the association between demographics and clinical data, such as age, treatment category, number of previous TB treatment cycles, and geopolitical zone, with gender. The independent effects of being a male or female DR-TB patient were determined using bivariate and multivariate analyzes with statistical significance of *p* < 0.05 and a 95% confidence interval. Records from a total of 2555 DR-TB patients were examined for the study. A majority were male (66.9%), largest age-group was 30–39 years old (35.8%), most had MDR-TB (61.4%), were HIV-negative (76.6%), and previously treated for TB (77.1%). The southwest treatment zone had the highest proportion of DR-TB patients (36.9%), and most DR-TB diagnoses occurred in 2016 (36.9%). On bivariate analysis, age, HIV status, treatment zone, and clinical patient group in DR-TB were significantly associated with male gender. On multivariate analysis, males aged 20–29 years (AOR: 0.19, 95% CI: 0.33–0.59, *p* = 0.001) and HIV-positive males (AOR: 0.44, 95% CI: 0.33–0.59, *p* = 0.001) had lower likelihood of MDR-TB as males in the south–south treatment zone (AOR: 1.88, 95% CI: 1.23–2.85, *p* = 0.03), and being male and aged ≥60 years (AOR: 2.19, 95% CI: 1.05–4.54, *p* = 0.036) increased the probability of DR-TB. The older male population from south–southern Nigeria and women of childbearing age had lower incidence of DR-TB than men of the same age. Tailored interventions to reduce HIV and DR-TB prevalence in the general population, particularly among women of childbearing potential, and treatment support for young and older men are relevant strategies to reduce DR-TB in Nigeria.

## 1. Introduction

Tuberculosis (TB) is a disease of public health concern, responsible for approximately 10 million new infections and 1.5 million deaths in 2018 [1]. Nigeria is one of the 30 countries with a high burden of TB, TB/HIV and drug-resistant TB (DR-TB) [2]. Several questions remain unanswered about how gender affects the epidemiology of TB. While gender differences in males and females are based on their biological differences, gender gives meaning to these biological differences based on socially constructed factors such as role, behavior, and expectations of each gender, which can influence TB epidemiology [3]. It is well documented in the literature that most reporting and survey data on TB prevalence report higher rates of TB in males than females [1]. However, this global average hides the heterogeneity between countries, regions, and cultures. Possible explanations for higher rates of TB in men include differences in biology (e.g., genetic makeup and sex hormones); differences in exposure to behavioral risk factors (e.g., smoking, alcohol, indoor air pollution and occupational exposure), as well as sociocultural factors such as stigma, income level and health-oriented behaviors [4,5]. Different effects of these factors on men and women often result in different pathways to TB diagnosis, care and treatment. TB is also the leading cause of death from infectious diseases in women worldwide [3]. Women make up 70% of the world’s poor and are disproportionately affected by HIV/AIDS [3]. These factors can limit their access to health care or increase the prevalence of TB. The socioeconomic differences in both genders also result in differential exposure to TB risk factors and TB disease progression. In low- and middle-income countries (LMICs) like Nigeria, women also have less access to health services and TB-specific services, suffer greater delays in TB detection and treatment, and suffer from atypical TB symptoms that are often overlooked by the health systems [3,6].

Many gender studies have focused on TB in general and have failed to assess the unique impact of gender on DR-TB in TB control programs. The few available reports in the literature are also inconclusive on how gender affects DR-TB [7]. It appears that in regions where previous TB posed a high risk for multidrug-resistant TB (e.g., Western Europe), men were more likely to develop multidrug-resistant TB (MDR-TB) than women, and vice versa in Eastern Europe, where primary DR-TB transmission was common [7]. This could mean that women are more consistent and less likely to receive inadequate TB treatment. Previous studies have also documented a higher proportion of MDR-TB in men [8,9]. This was explained by lower adherence, greater exposure to risk factors such as smoking and alcohol, and faster TB progression in men.

In South Africa, a significantly higher percentage of health-care workers (HCWs) admitted for MDR-TB or extensively drug-resistant TB (XDR-TB) were young and female, reflecting the massive HIV burden among South African women [10,11]. Nurses are known to be a high-risk group for nosocomial TB due to their direct and prolonged contact with TB patients [12]. Female frontline medical workers could therefore be more exposed to DR-TB from admitted patients or during DR-TB management in the community. Data from 81,794 TB patients of known gender also showed that female TB cases are 1.2 times more likely to harbor an MDR-TB strain than male TB cases [13]. In addition, women have more contact with sick MDR-TB patients at home. In Nigeria, two studies found no association between gender and DR-TB [14,15]. Although these few studies have focused on DR-TB, they only emphasize the unique risk of contracting the disease when one is male or female. However, these studies lacked age and other demographic and clinical information that might influence differences in acquiring the disease in men and women. The interaction of sex with DR-TB impacts different key populations in different settings. In this study, we examined the predictive factors influencing differences in acquiring DR-TB strains between males and females. Determining the predictive factors for both males and females to MDR-TB infection in a country or setting will therefore provide more insight into the epidemiology of the disease and help design targeted interventions and measures to improve access to care and reduce the risk of DR-TB disease.

### 1.1. Background

#### 1.1.1. DR-TB Model of Care in Nigeria

Nigeria has an estimated population of 202 million people and is divided into six geopolitical zones, with six states in each geopolitical zone and the Federal Capital Territory (FCT). There are two treatment models within the programmatic management of DR-TB in Nigeria, according to the National TB and Leprosy Control Program (NTBLCP) guidelines [2]. They are the hospital-based model and the community-based outpatient model. Nigeria began Programmatic Management of DR-TB (PMDT) in 2010 with hospital-based DR-TB care in a specialized treatment center at University College Hospital, Ibadan for the entire 8 months of the intensive phase, followed by community decentralization for outpatient services for the remaining 12 months of the continuation phase 2, under the supervision of NTBLCP [2]. However, since 2013, the NTBLCP has adopted the community outpatient DR-TB model for more effective and decentralized care of the growing number of DR-TB patients and in line with the 2011 WHO recommendation. In this new model, patients complete the entire 20 months of DR-TB treatment in an outpatient setting. In both the hospital and outpatient models, the management of the continuation phase is the same. A treatment support worker visits the patient’s home daily to administer the DOTS regimen, supplemented by biweekly visits to the patient at the DOTS center for medication collection. The local government authority (LGA) TB caregiver also makes a monthly home visit to the patient, while the state DR-TB team, a multidisciplinary team of experts, also visits the patient on a quarterly basis. The state DR-TB team consists of the state DR-TB contact person, a logistics officer, a state TB control officer, an ENT surgeon/audiometrist, a quality-assurance/laboratory officer, a social worker, a psychiatrist and a thoracic doctor, among other. Patients are matched to one of the two models by health-care professionals based on the patient’s medical history, the availability of the model in their geographic region, and the patient’s health status at the time of treatment initiation, taking into account the patient’s preference.

#### 1.1.2. DR-TB Case Finding and Diagnostic Service Coverage in Nigeria

The trend in reporting DR-TB cases has increased with the launch of GeneXpert as the number one diagnostic tool in 2016. The number of DR-TB cases increased by 35% from 1686 in 2016 to 2286 in 2017, although this accounted for only 11% of the estimated MDR/RR-TB cases [2]. Also, only 78% of diagnosed DR-TB cases were enrolled in DR-TB care in 2017 [2]. However, the DR-TB line listing tool later introduced by the NTBLCP improved accountability for each DR-TB case. The Global Fund through the Institute of Human Virology of Nigeria (IHVN) as the main beneficiary and the KNCV Tuberculosis Foundation through the USAID-funded Challenge TB (CTB) project are providing the main support for DR-TB diagnosis and treatment in Nigeria.

#### 1.1.3. DR-TB Treatment Coverage in Nigeria

The number of DR-TB treatment centers increased from 16 in 2016 to 27 in 2017 [2]. The goal of the National Council on Health (NCH), the highest health decision-making body in Nigeria, was to ensure the establishment of at least one DR-TB treatment center in each of the 36 states of the federation and FCT. Although there was at least one DR-TB treatment center in each geopolitical zone in 2017, only 70% of the states (26/36) had at least one DR-TB treatment center. Nonetheless, all the 36 states are now implementing community-based DR-TB. In 2017, the first large-scale drug-resistant TB (XDR-TB) unit was commissioned in southwestern Nigeria: five national reference laboratories upgraded to perform second-line LPA. Further, the use of a shorter DR-TB regimen was initiated while program and facility staff were trained in programmatic treatment of DR-TB (PMDT) and a shorter DR-TB regimen [2]. DR-TB challenges in Nigeria include inadequate local government (LGA) response to the provision of DR-TB services, delays in enrollment of diagnosed DR-TB patients, and suboptimal real-time data upload to the National Electronic TB Information Management Systems (NETIMS) platform by the LGA-TB supervisor and DR-TB treatment center staff [2].

## 2. Materials and Methods

### 2.1. Design

This was a retrospective cohort study using routinely collected national TB program data for Nigeria. The data were collected from the National TB and Leprosy Control Program database, which is the body for the repository of the data. Inclusion criteria were patients with drug-resistant TB between 2010 and 2016.

### 2.2. Data Collection and Analysis

Demographic and clinical information of all MDR-TB patients treated between July 2010 and December 2016 was extracted from the e-TB Manager-derived table prepared by the DR-TB focus staff of the National TB and Leprosy Control Program (NTBLCP). It was double-entered into Microsoft Excel version 2013, coded, cleaned, and imported into Statistical Package for Social Sciences (SPSS) version 20 for analysis. Descriptive statistics were used to analyze categorical variables from the patients’ sociodemographic and clinical characteristics, and are presented as frequencies and percentages in tables and graphs. Inferential statistics were used to explore the association of gender and the independent variables using the bivariate logistic regression model. Multivariate logistic regression was used to control for confounding variables. All odds ratios (ORs) and adjusted odds ratios (AORs) are presented with their 95% CIs and, a *p*-value ≤ 0.05 was considered statistically significant.

### 2.3. Variables Collected and Analyzed

The primary outcome variable was gender (male/female). Explanatory variables consisted of age (years), years of school enrollment, HIV status (positive, negative), DR-TB category (mono-DR, Rif-resistant, poly-DR and MDR), number of previous treatments (one, two, three or more), diagnosis type (bacteriologically confirmed, clinical), patient group (new, previously treated) and zone (NE, NW, NC, SE, SS, SW).

### 2.4. Ethics Approval

This study was approved by the National Health Research Ethics Committee of Nigeria (NHREC/01/01/2007), Jos University Teaching Hospital Ethics Committee (JUTH/DCS/ADM/127/XXIX/1586) and the Oyo State Research Ethics Review Committee (13/479/1370 AD). The study also met the Boston University Institutional Review Boards’ waiver criteria for analysis of routinely collected program data (H-38912). Patient information was anonymized and deidentified prior to analysis. Since the program data were routinely collected, the designated ethics committees approved the study and waived consent.

## 3. Results

### 3.1. Sociodemographic and Clinical Characteristics of TB Patients

**Figure 1:**
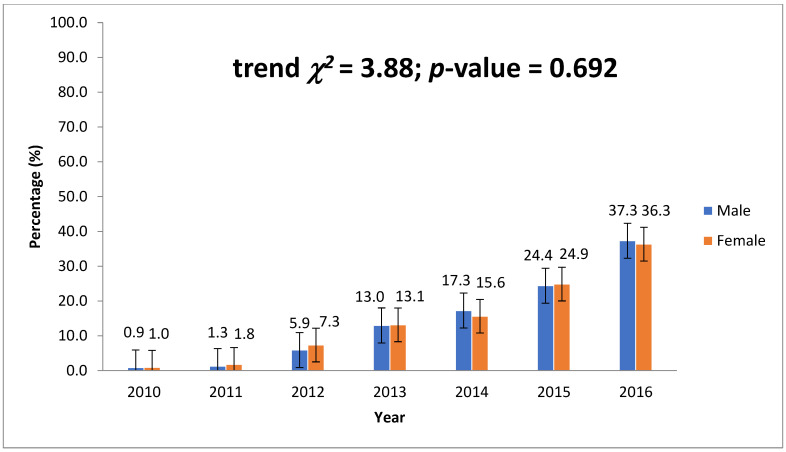
Trend of DR-TB cases by gender per year from 2010 to 2016.

Records from a total of 2555 patients were analyzed for the study. A majority of these patients (35.77%, 914) were between the ages of 30 and 39, followed by 20–29 (23.41%, 598), 40–49 (20.59%, 526), and 50–59 (8.61%, 220). Males represented 66.93% (1710) and HIV-positive 23.41% (305), which is the proportion of those with known HIV status from the study data. The proportion of patients with MDR-TB was 61.41% (1165) and mono-DR 27.78% (527). Almost half of the patients had had treatment twice (49.9%), and those with bacteriological diagnosis represented 88.78% (2240). Most patients diagnosed with MDR-TB (36.2%, 900) were in the southwest, followed by north–central and northwest, with 400 and 368 patients, respectively. The data show an increasing prevalence of DR-TB by year from 0.9% (23) in 2010 to 13.03% (333) in 2013 and 36.99% (945) in 2016, as shown in Table 1. The data show an increasing prevalence of DR-TB by year (Figure 1).

### 3.2. Results of Bivariate Analysis

The bivariate logistic regression model showed statistically significant associations with gender in DR-TB cases. These statistically significant associations were age, HIV status, current zone address, and patient group (Table 2).

Patients in the age categories 20–29 years (OR: 4.12, 95% CI: 2.52–673, *p* = 0.001) and 30–39 years (OR: 1.64, 95% CI: 1.09–2.48, *p* = 0.017) were four times and almost twice as likely, respectively, to be male patients experiencing DR-TB as those younger than 20 years or older than 39 years. Those previously treated (OR: 1.35, 95% CI: 1.11–1.64, *p* = 0.003) showed significantly increased odds of 1.35 compared to those new to treatment. Conversely, HIV-negative patients (OR: 0.53, 95% CI: 0.41–0.68, *p* = 0.001) and those in the south–south region (OR: 0.55–0.73, *p* = 0.001) were half as likely to be male patients experiencing DR-TB as shown in Table 2.

### 3.3. Gender as a Predictor of DR-TB

When adjusted for possible confounders, the multivariate logistic regression model showed statistically significant lower odds for men in aged 20–29 (AOR: 0.19, 95% CI: 0.09–0.40, *p* = 0.001) and higher for men in the south–south region (AOR: 1.88, 95% CI: 1.23–2.85, *p* = 0.003). HIV status was the same, and a new age range, 60 years and above, (60+) became significant with higher odds for men (AOR: 2.19, 95% CI: 1.05–4.54, *p* = 0.036), as shown in Table 3. However, patient group (AOR: 0.93, 95% CI: 0.68–1.26, *p* = 0.626) was no longer significant when other variables were adjusted.

## 4. Discussion

Our results showed that HIV-positivity and age 20–29 years reduced the likelihood of being a male MDR-TB carrier (compared to female MDR-TB patients). However, about 50% of the patients’ data for this study did not have their HIV status listed, which could have impacted our analysis. Also, being 60 years of age and in the south–south treatment zone (compared to the northeast zone) increased the likelihood of being a male MDR-TB patient.

Our results are similar to those of Tanzania, where a significantly higher proportion of female MDR-TB cases (61%) were aged 15–34 than males (45%), and India, where female MDR-TB-Patients were twice as likely to be in the 18–29 age-group compared to female patients in the same age-group [16]. A possible explanation for this finding is the higher prevalence of HIV in this age group. For example, in Nigeria, women in the 20–24 age-group have the highest gender HIV disparity for the same age-group and almost fourfold the HIV prevalence (1.3%) of men of the same age-group (0.4%) [17]. HIV increases the risk of TB progression by 20-fold and has been documented as a risk factor for primary DR-TB.

Our results contrast with studies in Ethiopia and northern India, which reported statistically higher male MDR-TB patients in the age-groups 24 years (77.2% versus 56.9%) and 21–45 years (versus females in the same age-group). It is difficult to compare these dichotomized and broader age categorizations with our study due to the lack of narrower age categories in our study [18,19]. This finding suggests that males, particularly the productive age-group of 20–29 years, are less affected by MDR-TB than females of the same age-group in Nigeria. Alternatively, MDR-TB may be underdiagnosed in this group. The impact of MDR-TB on families and communities in Nigeria is very severe in women of childbearing age. They take care of the family, play important social roles and are responsible for the children’s survival. Therefore, interventions to support their prompt diagnosis and treatment should be available at all levels. In addition, MDR-TB mortality is highest in women in their 20s to 30s, due to pregnancy [20] and discontinuation of TB treatment under the false belief that it will harm their unborn children and affect breastfeeding [21]. Therefore, strategies are needed to ensure a high index of suspicion so that MDR-TB can be promptly recognized and proven in women of childbearing age.

Based on our results, male MDR-TB patients were more likely to be >65 years old than females. This result is similar to a report on gender assessment of DR-TB in Kenya, with a significantly higher DR-TB burden in males >65 years than in females of similar age [22]. In general, TB occurs more frequently in female in pre-adolescence and puberty and becomes equal or slightly more common than male in the reproductive age-group [3,22]. Reports have shown that TB incidence increases with age in men and is higher than women of the same age-group throughout the age bands, similar to what was found in our study. The noncompliance of male DR-TB patients (compared to females), which has been described earlier, and males being more likely to require retreatment due to failure to comply could be some of the explanations for this phenomenon [23,24,25]. This finding is of critical importance, because in contrast to women, where TB mortality is highest in the 20–30 age-group, TB mortality in men peaks much later in life [26]; therefore active searching for DR-TB cases in older men needs to be intensified and DR-TB medication promptly started.

The results of an increased likelihood of male DR-TB in patients from the south–south zone treatment center could mean a higher risk of treatment failure, increasing the risk of MDR-TB in men from this zone. A previous study from southern Nigeria reported higher conversion failures after two months and five months of TB treatment in males than females. In this study, men were also twice as likely to fail TB treatment [27]. These results may explain why men from the south–south treatment zone in our study were at increased risk of DR-TB.

Similarly, to our findings, several studies have identified HIV as a risk factor of MDR-TB, although not specifying how it varies between males and females [28,29,30]. HIV had no association with DR-TB in some studies [31,32,33]. However, in France and Ukraine, HIV was associated with primary DR-TB, but not with secondary DR-TB in France [34,35]. Our findings that HIV-negative status reduced the likelihood of male MDR-TB are consistent with what is obtainable in the literature [36]. We found that the association between male gender and increased DR-TB is largely moderated by a high risk of prior TB treatment in males. In support of this, although more men than women belonged to a previously treated category (88% vs. 81%), a study from Ethiopia found no association between HIV serostatus and gender [18]. This may be due to the higher HIV prevalence in males in that study, in contrast to our setting, where HIV prevalence was higher in females. People living with HIV may also be more exposed to DR-TB in correctional facilities due to an increased likelihood of hospitalization in settings with suboptimal infection control or exposure to peers who may have DR-TB.

## 5. Conclusions

Our study found that increased DR-TB diagnoses among young women aged 20–29 in Nigeria are primarily due to higher HIV prevalence in this population when compared to male peers of the same age. This is most likely due to intergenerational relationships and a culture in Nigeria that encourages young girls to marry much older men. Men over the age of 50 have the highest HIV burden in Nigeria, and dating much younger women increases intergenerational transmission of HIV and the female population’s risk of DR-TB. According to a 2017 survey, 41.2% of women aged 15–24 had had a sexual partner who was 10 or more years older than them in the previous 12 months [37]. This raises the risk of HIV in this population because the virus is frequently transmitted from older men to younger women. Integrated screening for DR-TB and HIV at the facility and community levels for this population of women will be a cost-effective intervention for early detection and treatment, given the disease’s impact not only on the woman but also on child survival. Policies that assist older males, primarily in the south–south zone, should be supported to complete treatment and reduce the risk of DR-TB. Innovative programs that address the social and economic determinants and barriers that women face when seeking TB treatment should be included in the country’s TB strategic plan and evaluated in future studies. This study’s data are primarily on DR-TB, with little information on TB epidemiology in Nigeria. Furthermore, there are no sociodemographic data on DR-TB, such as occupation, educational status, level, and type of employment. Other contexts such as smoking and alcohol consumption and health-system factors, such as health-care worker attitudes, accessibility and proximity to health-care centers, availability of first- and second-line anti-tuberculosis medications, and so on were also unavailable. All of these are limitations of this study, and further research in this area will shed more light on the subject.

## Figures and Tables

**Table 1:** DR-TB patients characteristics in Nigeria (2010–2016) (*n = 2555*).

Variables	Frequency (n)	Percentage (%)	*df*	*χ^2^ (p-Value)*
**Age (years)**				
≤19	161	6.30	5	1337.11 (0.001) *
20–29	598	23.41		
30–39	914	35.77		
40–49	526	20.59		
50–59	220	8.61		
60+	136	5.32		
**Gender**				
Female	845	33.07	1	585.69 (0.001) *
Male	1710	66.93		
HIV Status **				
Positive	305	23.41	1	1445.70 (0.001) *
Negative	998	76.59		
DR-TB category **				
Mono-DR	527	27.78	3	2904.72 (0.001) *
RIF-Resistant	176	9.28		
Poly-DR	29	1.53		
MDR	1165	61.41		
Number of previous Treatments **				
One	302	36.70	2	787.60 (0.001) *
Two	411	49.94		
3+	110	13.37		
Diagnosis type **				
Bacteriological confirmed	2240	88.78	1	3071.91 (0.001) *
Clinical	283	11.22		
Zone current address **				
NE	221	9.06	5	1012.0 (0.001) *
NW	368	15.09		
NC	400	16.41		
SE	195	8.00		
SS	354	14.52		
SW	900	36.92		
Patient group **				
New	567	22.89	1	1501.55 (0.001) *
Previously treated	1910	77.11		
**Year**				
2010	23	0.90	6	2158.0 (0.001) *
2011	37	1.45		
2012	163	6.38		
2013	333	13.03		
2014	427	16.71		
2015	627	24.54		
2016	945	36.99		

* Statistically significant (*p* < 0.05), df = degrees of freedom. ** Incomplete data and not summing to *n* = 2555, M:F is 2:1.

**Table 2:** Association of gender and DR-TB cases in Nigeria (2010–2016) (*n = 2555)* (bivariate).

Variables	Gender		OR (95 CI)	*p-Value*
	Male (*n* = 1710)	Female (*n* = 845)		
	Freq (%) [95CI]	Freq (%) [95CI]		
**Age (years)**				
≤19 ^R^	62 (38.51)	99 (61.49)	*Ref*	
20–29	365 (61.04)	233 (38.96)	4.12 (2.52–6.73)	0.001 *
30–39	652 (71.33)	262 (28.67)	1.64 (1.09–2.48)	0.017 *
40–49	364 (69.20)	162 (30.80)	1.04 (0.69–1.55)	0.862
50–59	169 (76.82)	51 (23.18)	1.15 (0.76–1.74)	0.518
60+	98 (72.06)	38 (27.94)	0.78 (0.48–1.27)	0.314
HIV Status **				
Positive	162 (53.11)	143 (46.89)	*Ref*	
Negative	681 (68.24)	317 (31.76)	0.53 (0.41–0.68)	0.001 *
DR-TB category **				
Mono-DR ^R^	349 (66.22)	178 (33.78)	*Ref*	
RIF-Resistant	120 (68.18)	56 (31.82)	1.12 (0.90–1.39)	0.302
Poly-DR	18 (62.07)	11 (37.93)	1.03 (0.73–1.44)	0.878
MDR	801 (68.76)	364 (31.24)	1.35 (0.63–2.88)	0.445
Number of previous Treatments **				
One ^R^	183 (60.60)	119 (39.40)	*Ref*	
Two	280 (68.13)	131 (31.87)	1.34 (0.84–2.12)	0.217
3+	74 (67.27)	36 (32.73)	0.96 (0.61–1.51)	0.865
Diagnosis type **				
Bacteriologically confirmed	1495 (66.74)	745 (33.26)	*Ref*	
Clinical	193 (68.20)	90 (31.80)	0.94 (0.72–1.23)	0.672
Zone current address **				
NE ^R^	154 (69.68)	67 (30.32)	*Ref*	
NW	281 (76.36)	87 (23.64)	0.84 (0.65–1.08)	0.162
NC	272 (68.0)	128 (32.0)	0.77 (0.56–1.06)	0.113
SE	122 (62.56)	73 (37.43)	0.95 (0.73–1.23)	0.676
SS	231 (66.96)	123 (33.04)	0.55 (0.42–0.73)	0.001 *
SW	576 (64.0)	324 (36.0)	1.06 (0.77–1.45)	0.705
Patient group **				
Previously treated	1306 (68.38)	604 (31.62)	*Ref*	
New	349 (61.55)	218 (38.45)	1.35 (1.11–1.64)	0.003 *

* Statistically significant (*p* < 0.05) R = Reference. ** Incomplete data and not summing to *n* = 2555.

**Table 3:** Association of gender and DR-TB cases in Nigeria (2010–2016) (*n = 2555*) (Multivariate).

Variables	Gender		AOR (95 CI)	*p-Value*
	Male (*n* = 1710)	Female (*n* = 845)		
	Freq (%) [95CI]	Freq (%) [95CI]		
**Age (years)**				
≤19 ^R^	62 (38.51)	99 (61.49)	*Ref*	
20–29	365 (61.04)	233 (38.96)	0.19 (0.09–0.40)	0.001 *
30–39	652 (71.33)	262 (28.67)	0.63 (0.35–1.14)	0.628
40–49	364 (69.20)	162 (30.80)	1.31 (0.73–2.35)	0.366
50–59	169 (76.82)	51 (23.18)	1.19 (0.65–2.18)	0.584
60+	98 (72.06)	38 (27.94)	2.19 (1.05–4.54)	0.036 *
HIV Status **				
Positive	162 (53.11)	143 (46.89)	*Ref*	
Negative	681 (68.24)	317 (31.76)	0.44 (0.33–0.59)	0.001 *
Zone current address **				
NE ^R^	154 (69.68)	67 (30.32)	*Ref*	
NW	281 (76.36)	87 (23.64)	1.34 (0.94–1.93)	0.111
NC	272 (68.0)	128 (32.0)	1.13 (0.68–1.90)	0.636
SE	122 (62.56)	73 (37.43)	0.88 (0.60–1.29)	0.511
SS	231 (66.96)	123 (33.04)	1.88 (1.23–2.85)	0.003 *
SW	576 (64.0)	324 (36.0)	0.77 (0.49–1.19)	0.242
Patient group **				
Previously treated	1306 (68.38)	604 (31.62)	*Ref*	
New	349 (61.55)	218 (38.45)	0.93 (0.68–1.26)	0.626

* Statistically significant (*p* < 0.05) R = Reference, ** Incomplete data and not summing to *n* = 2555.

## Data Availability

The datasets generated and analyzed during the current study are not publicly available. Data are, however, available from the authors upon reasonable request and with permission of the National Tuberculosis, Leprosy, and Buruli Ulcer Control Program (NTBLCP).

## References

[1] World Health Organization (2018). Global Tuberculosis Control: WHO Report 2018.

[2] Federal Ministry of Health [FMOH], Department of Public Health (2018). National TB and Leprosy Control Programme Annual Report.

[3] Hof S., Najlis C., Bloss E., Straetema M. A Systematic Review on the Role of Gender in Tuberculosis Control. https://www.kncvtbc.org/uploaded/2015/09/Role_of_Gender_in_TB_Control.pdf.

[4] Borgdorff M.W., Nagelkerke N.J., Dye C., Nunn P. (2000). Gender and tuberculosis: A comparison of prevalence surveys with notification data to explore gender differences in case detection. Int. J. Tuberc. Lung Dis..

[5] Uplekar M., Rangan S., Ogden J. (1999). Gender and Tuberculosis Control: Towards a Strategy for Research and Action. WHO/TB/2000.

[6] Adejumo O.A., Daniel O.J., Otesanya A.F., Adejumo E.N. (2016). Determinants of health system delay at public and private directly observed treatment, short course facilities in Lagos State, Nigeria: A cross-sectional study. Int. J. Mycobacteriol..

[7] Faustini A., Hall A., Perucci C. (2006). Risk factors for multidrug resistant tuberculosis in Europe: A systematic review. Thorax.

[8] Moss A.R., Alland D., Telzak E., Hewlett D., Sharp V., Chiliade P., LaBombardi V., Kabus D., Hanna B., Palumbo L. (1997). A city-wide outbreak of a multiple-drugresistant strain of Mycobacterium tuberculosis in New York. Int. J. Tuberc. Lung Dis..

[9] Edlin B.R., Tokars J.I., Grieco M.H., Crawford J.T., Williams J., Sordillo E.M., Ong K.R., Kilburn J.O., Dooley S.W., Castro K.G. (1992). An outbreak of multidrug-resistant tuberculosis among hospitalised patients with the acquired immunodeficiency syndrome. N. Engl. J. Med..

[10] O’Donnell M.R., Jarand J., Loveday M., Padayatchi N., Zelnick J., Werner L., Naidoo K., Master I., Osburn G., Kvasnovsky C. (2010). High Incidence of Hospital Admissions with Multidrug Resistant and Extensively Drug Resistant Tuberculosis among South African Health Care Workers. Ann. Intern. Med..

[11] Abdool Karim S.S., Churchyard G.J., Karim Q.A., Lawn S.D. (2009). HIV infection and tuberculosis in South Africa: An urgent need to escalate the public health response. Lancet.

[12] Joshi R., Reingold A.L., Menzies D., Pai M. (2006). Tuberculosis among healthcare workers in low- and middle-income countries: A systematic review. PLoS Med..

[13] World Health Organization (2009). Global Tuberculosis Control–Epidemiology, Strategy, Financing.

[14] Olusoji D., Elutayo O., Olanrewaju O., Olapade G.D. (2013). Pre-extensive drug resistant TB among MDR-TB patients. Global Advd. Res. J. Microbiol..

[15] Lawson L., Yassin M.A., Abdurrahman S.T., Parry C.M., Dacombe R., Sogaolu O.M., Ebisike J.N., Uzoewulu G.N., Emenyonu N., Ouoha J.O. (2011). Resistance to first-line tuberculosis drugs in three cities of Nigeria. Trop. Med. Int. Health.

[16] Begum V., De Colombani P., Das Gupta S., Salim A.H., Hussain H., Pietroni M., Rahman S., Pahan D., Borgdorff M.W. (2001). Tuberculosis and patient gender in Bangladesh: Sex differences in diagnosis and treatment outcome. Int. J. Tuberc. Lung Dis..

[17] (2019). National HIV/AIDS Indicator and Impact Survey (NAIIS). https://www.naiis.ng/resource/factsheet/NAIIS%20PA%20NATIONAL%20FACTSHEET%20FINAL.pdf..

[18] Worku Y., Getinet T., Mohammed S., Yang Z. (2018). Drug-Resistant tuberculosis in Ethiopia: Characteristics of cases in a referral hospital and the implications. Int. J. Mycobacteriol..

[19] Anupurba S., Sinha P., Srivastava G.N., Gupta A. (2017). Association of risk factors and drug resistance pattern in tuberculosis patients in North India. J. Glob. Infect. Dis..

[20] Mathad J., Gupta A. (2012). Tuberculosis in pregnant and postpartum women: Epidemiology, management, and research gaps. Clin. Infect. Dis..

[21] Hudelson P. (1996). Gender Differentials in Tuberculosis: The Role of Socio-economic and Cultural Factors. Tuber. Lung Dis..

[22] (2018). Stop TB Partnership. http://www.stoptb.org/assets/documents/communities/CRG/TB%20Gender%20Assessment%20Kenya.pdf..

[23] Chan-Yeung M., Noertjojo K., Leung C.C., Chan S.L., Tam C.M. (2003). Prevalence and predictors of default from tuberculosis treatment in Hong Kong. Hong Kong Med. J..

[24] Samman Y., Krayem A., Haidar M., Mimesh S., Osoba A., Al-Mowaallad A., Abdelaziz M., Wali S. (2003). Treatment outcome of tuberculosis among Saudi nationals: Role of drug resistance and compliance. Clin. Microbiol. Infect..

[25] Oliveira H., Moreira F. (2000). Treatment abandonment and tuberculosis recurrence: Aspects of previous episodes, Brazil, 1993–1994. Rev. Saúde Pública.

[26] Watkins R., Plant A. (2006). Does smoking explain sex differences in the global tuberculosis epidemic?. Epidemiol. Infect..

[27] Oshi S.N., Alobu I., Ukwaja K.N., Oshi D.C. (2015). Investigating gender disparities in the profile and treatment outcomes of tuberculosis in Ebonyi state, Nigeria. Epidemiol. Infect..

[28] Yimer S.A., Agonafir M., Derese Y., Sani Y., A Bjune G., Holm-Hansen C. (2012). Primary drug resistance to anti-tuberculosis drugs in major towns of Amhara region, Ethiopia. APMIS-Acta Pathol. Microbiol. Immunol. Scand..

[29] Abebe G., Abdissa K., Abdissa A., Apers L., Agonafir M., De-Jong B.C., Colebunders R. (2012). Relatively low primary drug resistant tuberculosis in southwestern Ethiopia. BMC Res. Notes.

[30] Abate D., Taye B., Abseno M., Biadgilign S. (2012). Epidemiology of anti-tuberculosis drug resistance patterns and trends in tuberculosis referral hospital in Addis Ababa, Ethiopia. BMC Res. Notes.

[31] Hirpa S., Medhin G., Girma B., Melese M., Mekonen A., Suarez P., Ameni G. (2013). Determinants of multi drug- resistant tuberculosis in patients who underwent first-line treatment Addis Ababa: A case-control study. BMC Public Health.

[32] Mesfin M., Tasew W., Richard J. (2005). The quality of tuberculosis diagnosis in districts of Tigray region of northern Ethiopia. Ethiop. J. Health Dev..

[33] Flora M.S., Amin M.N., Karim M.R., Afroz S., Islam S., Alam A., Hossain M. (2013). Risk factors of multidrug resistant tuberculosis in Bangeladeshi population: A case control study. Bangladesh Med. Res. Counc. Bull..

[34] Akksilp S., Wattanaamornkiat W., Kittikraisak W., Nateniyom S., Rienthong S., Sirinak C., Ngamlert K., Mankatittham W., Sattayawuthipong W., Sumnapun S. (2009). Multidrug resistant TB and HIV in Thailand: Overlapping, but not independently associated, risk factors. Southeast Asian J. Trop. Med. Public Health.

[35] Schwoebel V., Decludt B., De Benoist A.-C., Haeghebaert S., Torrea G., Vincent V., Grosset J. (1998). Multidrug resistant tuberculosis in France 1992–4: Two case-control studies. BMJ.

[36] Baluku J.B., Mukasa D., Bongomin F., Stadelmann A., Nuwagira E., Haller S., Ntabadde K. (2021). Gender differences among patients with drug resistant tuberculosis and HIV co-infection in Uganda: A countrywide retrospective cohort study. BMC Infect. Dis..

[37] United Nations Children Emergency Fund UNICEF Data: Monitoring the Situation of Children and Women 2017. https://data.unicef.org/topic/hivaids/global-regional-trends/.

